# *In-vitro* release pharmacokinetics of amikacin, teicoplanin and polyhexanide in a platelet rich fibrin—layer (PRF)—a laboratory evaluation of a modern, autologous wound treatment

**DOI:** 10.1371/journal.pone.0181090

**Published:** 2017-07-07

**Authors:** Daniela Knafl, Florian Thalhammer, Matthias G. Vossen

**Affiliations:** Division of Infectious Diseases and Tropical Medicine, Department of Medicine I, Medical University of Vienna, Vienna, Austria; Christian Albrechts Universitat zu Kiel, GERMANY

## Abstract

**Objectives:**

Platelet rich fibrin (PRF) is an autologous fibrin glue, produced from patients' blood, which, besides intraoperative use, has applications in the treatment of infected wounds. The combination with antimicrobial agents results in a prolonged antibacterial effect allowing for wound dressing change intervals of seven days even in infected wounds. The aim of this study was to evaluate release kinetics of amikacin, teicoplanin or polyhexanide from a PRF-layer.

**Methods:**

PRF mixed with teicoplanin, amikacin or polyhexanide was sprayed on a silicon gauze patch and put on a colombia agar with bacteria with known minimal inhibitory concentration (MIC) and incubated for 24 hours and afterwards transferred to another agar with the same bacterial strain. Inhibition zones were measured every 24 hours. This was repeated on 7 consecutive days. Antibiotic concentrations were calculated by interpolation.

**Results:**

More than 1000 mg/L teicoplanin were released within the first 24 hours and 28.22 mg/L after 168 hours. Amikacin release was above 10,000 mg/L within the first 24 hours and still 120.8 mg/L after 120 hours. A release of polyhexanide could be verified for the first 24 hours only. Consequently teicoplanin and amikacin released from PRF showed antimicrobial *in-vitro* effects for almost a week, whereas an antimicrobial effect of polyhexanide could only be verified for the first 24 hours.

**Conclusions:**

Our Results show that a weekly dressing regimen may be justified in wounds treated with PRF plus amikacin or teicoplanin, since bacteria will be eradicated over a considerable period of time after a single application of PRF.

## Introduction

**Fig 1:**
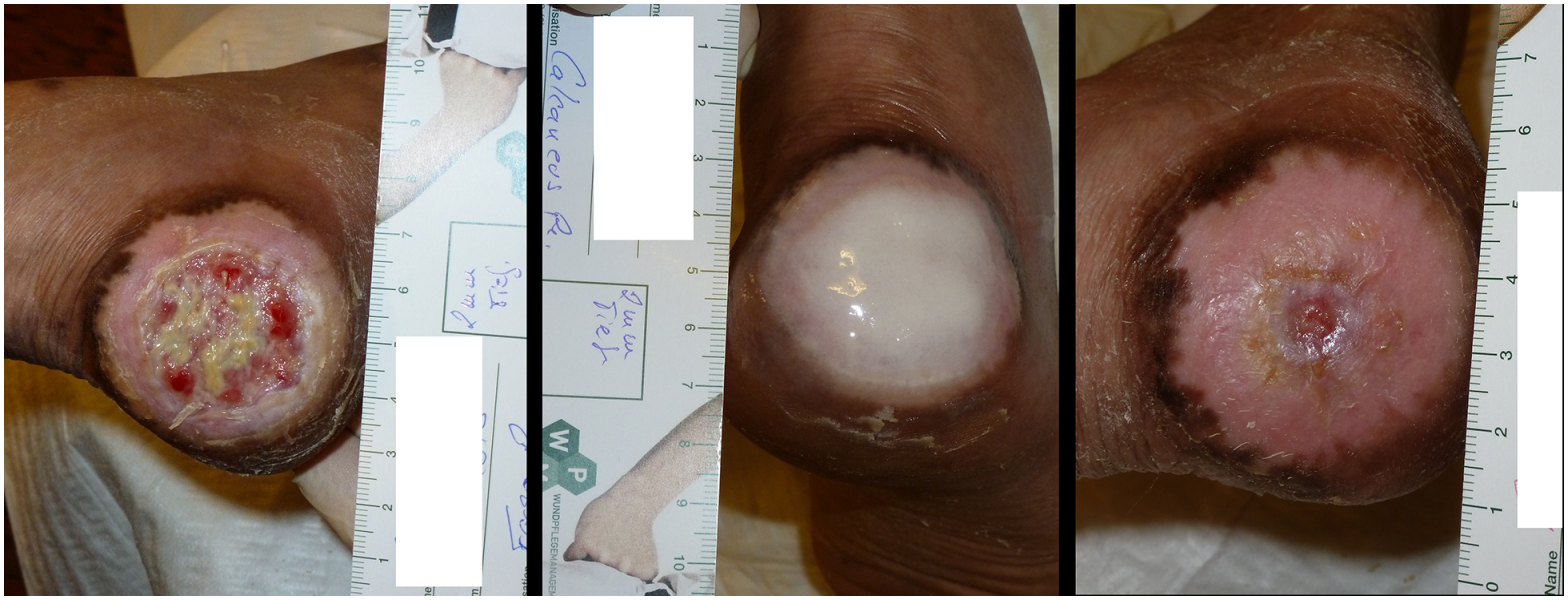
Course of chronic acral heel—ulcer. A: initial wound without PRF-treatment (diameter of wound 6 cm), B: PRF and amikacin applied to wound, C: 11 weeks after initiation of therapy and after 10 applications of PRF and amikacin (diameter of wound 0.75 cm).

Prevention and treatment of wound infections is an important topic in surgery as well as in the management of wounds. Chronic leg ulcers, which are caused by a variety of entities, are a common health issue worldwide. [1] They are known for their poor ability to heal, leading to patients suffering and remarkable treatment costs. [2] Fibrin and platelets serve as important components in wound healing. Especially fibrin highly determinates the healing process by formation of a cohesive network, which provides a temporary support to wound healing. Furthermore it actively enhances cell migration, adhesion and tubule formation leading to cell growth, angiogenesis and antimicrobial effects by recruitment of proinflammatory cells. [3] Physiologically fibrin and platelets are released as a reaction to trauma, which is the case after obtaining a wound or after debridement. However, in patients with surgical wound complications or chronic ulcers, the self-healing abilities of the human body do not suffice to allow for physiologic wound healing. Platelet rich fibrin (PRF) is a homologous concentrate produced from patients' blood, containing platelets in a fibrin matrix, which has shown to lead to significant improvement of chronic wounds previously not responding to therapy. [2,4] The positive effects of platelet-derivatives have already been described in the past. It could be shown that platelet-released growth factor (PRGF), which is a mixture of autologous proteins and growth factors prepared from platelet rich plasma (PRP), up-regulates endogenous vascular endothelial growth factor (VEGF) and prevents oxidative damage through the activation of a system of detoxifying and antioxidative enzymes. This activation occurs via induction of nuclear factor-like2 (Nrf2), which plays an important role in the transcriptional activation of antioxidant response element (ARE)-genes. [5,6] Although PRF does show an antibacterial effect, there are no data, that PRF-monotherapy has benefits in the treatment of infected wounds, but infections seem to impair the healing process. [7–10] Therefore, although compelling data is lacking, in treatment or prophylaxis of infected wounds PRF is often combined with antiseptics, such as polyhexanide, or antibiotics, such as vancomycin, teicoplanin, gentamicin or amikacin with the intent to eradicate bacteria and increase the healing process. In the clinical setting it is common that dressings of infected wounds, treated with PRF, are changed once a week only. A vivid patient example is given in Fig 1 (Fig 1). It displays the course of disease of a patient suffering from a chronic heel ulcer treated in our outpatient department in 2013, receiving PRF with amikacin as local treatment once a week due to stagnation of the ulcer under standard wound management. In several wound swabs growth of a fully susceptible *Pseudomonas aeruginosa* could be shown. Within 11 weeks and 10 applications of a PRF-amikacin-mixture a significant reduction in wound size was achieved. Prolonged dressing change intervals lead to a significant cost reduction without influencing wound healing negatively, which is a considerable reason for the implementation of a once weekly dressing change. [11] To further back the clinical observation that a weekly treatment suffices even in infected wounds, the release kinetics of teicoplanin, amikacin and polyhexanide were analysed.

## Materials and methods

**Fig 2:**
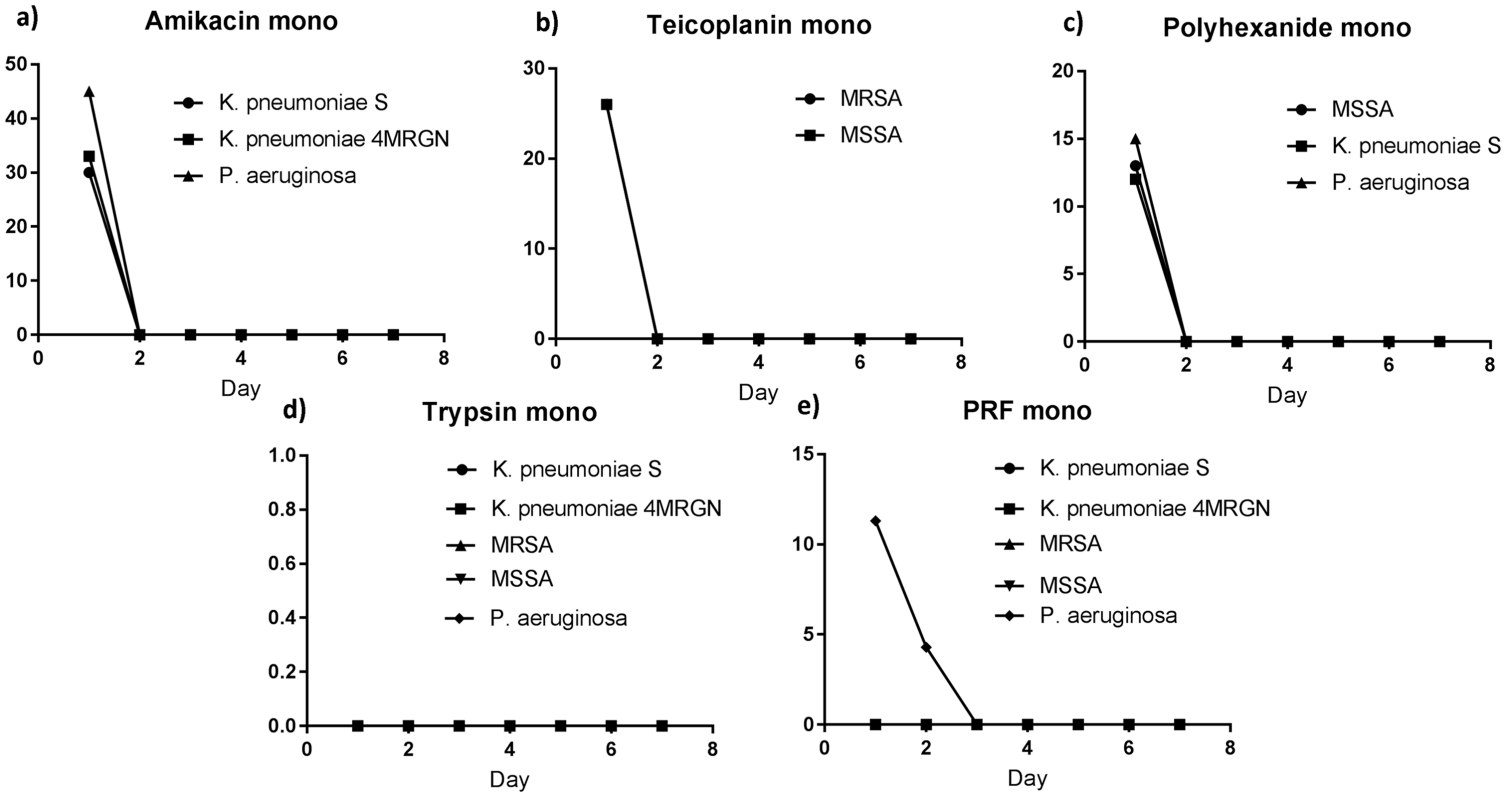
a) Positive controls amikacin-only, b) Positive controls teicoplanin-only, c) Positive controls polyhexanide-only, d) Negative controls trypsin-only, e) Negative controls PRF-only.

The study was conducted as prospective *in-vitro* study. The Ethics Committee of the Medical University of Vienna approved the conduct of the study (EC number: 1617–2015). Five volunteers donated blood for the production of PRF. After giving written and oral informed consent, a blood test was performed to examine the volunteer`s haemoglobin-level. If haemoglobin was > 10 g/dl, 240 ml blood was drawn for PRF- production. PRF was produced in a closed, two compartment cartridge by centrifugation after addition of sodiumcitrate and tranexamic acid (Vivostat^®^ PRF Preparation Device, Vivostat^®^ A/S, Denmark). During centrifugation thrombocytes and plasma were transported to a secondary chamber where batroxobin and pH 4 buffers were added. The resulting platelet rich fibrin solution was then injected into a syringe connected to the PRF production cartridge. The syringe was removed from the cartridge and the resulting PRF was then split into aliquots containing approximately 0.5 ml. Each aliquot was frozen at minus 20°C. 0.5 ml of PRF were co-delivered in a 1:1 ratio with antimicrobial agent using a PRF delivery system (Applicator Unit/ Co-Delivery Applicator Vivostat^®^ A/S, Denmark) on a 1 cm^2^ piece of silicon gauze (Sorbion^®^ plus, Sorbion^®^, Austria). For PRF application a micro scale was used to ensure that exactly 0.5 ml were delivered onto the patch. Amikacin (250 mg/ml), teicoplanin (125 mg/ml) and polyhexanide served as antimicrobial co-delivered substances. Dosing of antibiotics was chosen according to doses commonly used in the clinical setting. To simulate in-vivo PRF degradation by serine proteases, 2 μg trypsin 0.05% in EDTA were added. [12] Columbia-full-blood-agars were prepared with methicillin-sensitive *Staphylococcus aureus* (MSSA ATCC 29213), methicillin-resistant *Staphylococcus aureus* (MRSA ATCC 33592), *Pseudomonas aeruginosa* (*P*. *aeruginosa* ATCC 27853), *Klebsiella pneumoniae* (*K*. *pneumoniae* S), and *K*. *pneumoniae* 4MRGN all with a bacterial suspension density of 0.5 McFarland. Minimal inhibitory concentrations (MIC) of each bacterial strain were acquainted prior and double-checked with E-tests on 5 different days. (Table 1) The PRF loaded silicone gauze was put on these Columbia-full-blood-agars and incubated for 24 hours. (Table 2) After 24 hours the patch was transferred to another agar with the same bacterial strain. This was repeated on 7 consecutive days. Inhibition zone sizes were measured with a ruler every 24 hours before the transfer of the patch. Incubation of the bacterial strains with PRF-only and trypsin-only served as negative controls (Fig 2), and teicoplanin-, amikacin- and polyhexanide loaded control patches (Assay Discs, Schleicher & Schuell^®^) served as positive controls (Fig 2). Previous to application, the blank control-patches were incubated on a blank Colombia-full-blood-agar to exclude the risk of contamination. Each experiment was performed in triplicate. Furthermore, standard curves with serial dilutions from 1024 to 32 mg/L of each antibiotic in the presence of PRF were performed to assess potential effects of PRF on the antimicrobial activity, using the methods outlined above. However, for this experiment only 100 μg of PRF plus co-delivery substance were used per patch due to the considerable amount of patches and thus blood donations needed for this experiment. Descriptive statistics were performed using Microsoft^®^ Excel:Mac 2011 and GraphPad Prism 6^®^ for Mac. Antimicrobial concentrations were calculated via interpolation by means of the results of the positive controls using GraphPad Prism^®^. A non-linear regression model was chosen, using the least squares method with a hyperbola (X is concentration) as standard curve. Means of positive controls with concentrations in quadratic serial dilutions served as standards.

**Table 1:** Used bacterial strains with MIC-range [13].

Bacterial strain	MIC range (substance)
MSSA ATCC 29213	0.25–1 (teicoplanin)
MRSA ATCC 33592	0.25–1 (teicoplanin)
*K*. *pneumoniae* S	2–4 (amikacin)
*K*. *pneumoniae* 4MRGN	4–8 (amikacin)
*P*. *aeruginosa* ATCC 27853	1–4 (amikacin)

**Table 2:** Incubation of bacteria with PRF ± antimicrobial agent—Methods.

”Treatment”	MSSA	*K*. *pneumoniae*	*P*. *aeruginosa*	MRSA	*K*. *pneumoniae* 4MRGN
**PRF + Teicoplanin (125 mg/ml)**	X			X	
**PRF + Amikacin (250 mg/ml)**		X	X		X
**PRF + Polyhexanide/Macrogol**	X	X	X		
**PRF (negative control)**	X	X	X	X	X
**Trypsin 0,05% (neg. control)**	X	X	X	X	X
**Teicoplanin (positive control)**	X			X	
**Amikacin (positive control)**		X	X		X
**Polyhexanide (positive control)**	X	X	X		

## Results

### PRF plus teicoplanin

**Fig 3:**
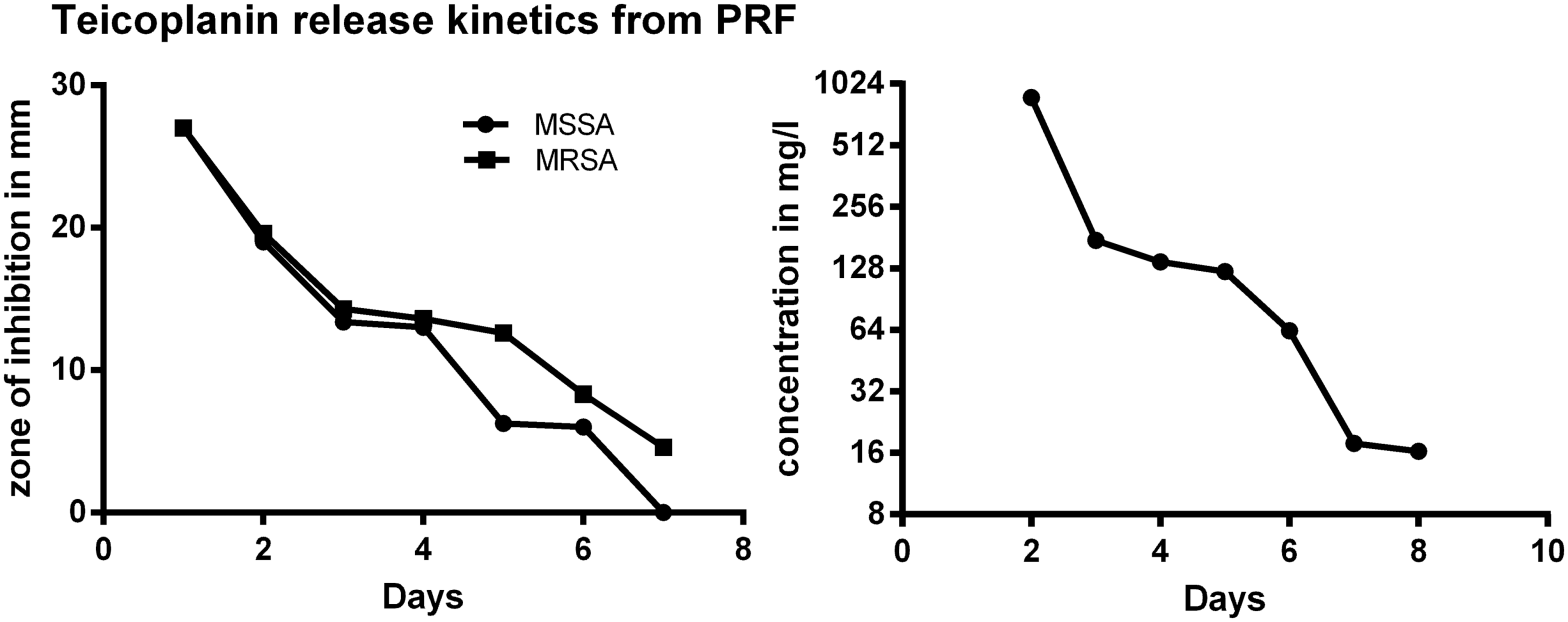
Left: Mean zones of inhibition for MSSA and MRSA incubated with PRF and teicoplanin (MSSA σ = 0–5.5 mm; MRSA σ = 0–11.43 mm). Right: Release of teicoplanin out of a PRF-layer (σ = 7.5–42.8 mg/L; R square = 0.81).

Zones of inhibition of MSSA were measurable until day 6 (144 h) and those of MRSA until day 7 (168 h) after the first incubation. The mean value was calculated of all triplet samples for the inhibition zone sizes of each bacterial strain each day (MSSA σ = 0–5.5 mm; MRSA σ = 0–11.43 mm) (Fig 3). Controls with trypsin only and PRF only remained negative. Teicoplanin release out of the PRF-layer showed concentrations of > 1000 mg/L after 25 hours of incubation, 875.07 mg/L after 48 hours and 17.79 mg/L (σ = 14.11 mg/L) after 168 hours (day 7). Standard deviation ranged because of the variation of inhibition zone sizes. (σ = 7.5–42.8 mg/L; R square = 0.81) (Fig 3).

Standard curves for teicoplanin in different concentrations in the presence of PRF did not show consistency, and could not point towards synergistic effects of teicoplanin and PRF (S1 Fig).

### PRF plus amikacin

**Fig 4:**
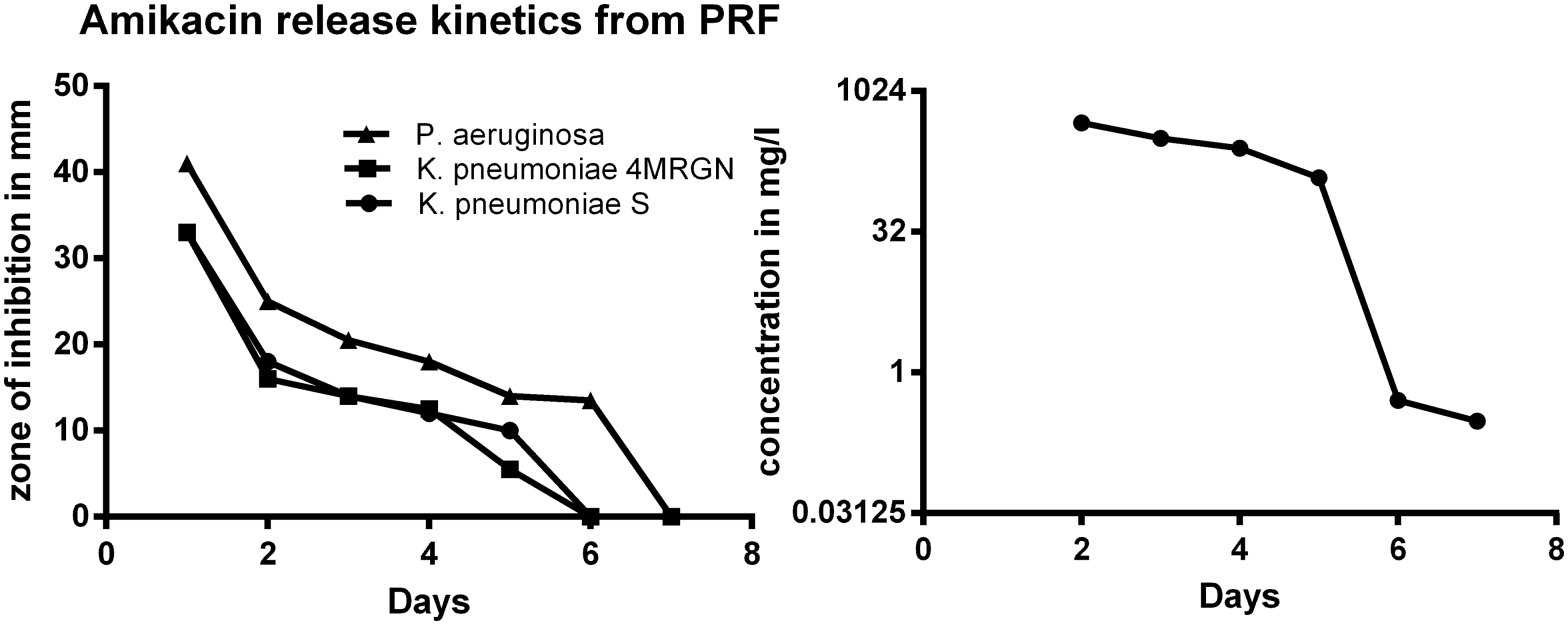
Left: Mean zones of inhibition for gram-negative bacteria with PRF and amikacin (*K*. *pneumoniae* S σ = 0 mm; *K*. *pneumoniae* 4MRGN σ = 5.5 mm). Right: Release of amikacin out of a PRF- layer.

Inhibition zones were measurable for *K*. *pneumoniae* S and *K*. *pneumoniae* 4MRGN until day 5 (120 h), and for *P*. *aeruginosa* until day 6 (144 h). Standard deviation ranged between σ = 0 mm for *K*. *pneumoniae* S, to σ = 5.5 mm for *K*. *pneumoniae* 4MRGN (Fig 4). *P*. *aeruginosa* had to be excluded from the interpolation-process and thereby was not considered for the calculation of amikacin release kinetics because PRF-only controls were positive. PRF—only on *P*. *aeruginosa* lead to an inhibition zone size of 13 mm within the first 24 hours of incubation. Amikacin release from PRF was demonstrable until day 5 (120 hours). Within the first 24 hours of incubation concentrations > 10,000 mg/L amikacin were released, on day 5 there was still a release of 120.8 mg/L amikacin (σ = 31.44–43.43 mg/L; R square = 0.99) (Fig 3).

Standard curves for amikacin in different concentrations in the presence of PRF could not point towards synergistic effects of amikacin and PRF (S2 Fig).

### PRF plus polyhexanide

**Fig 5:**
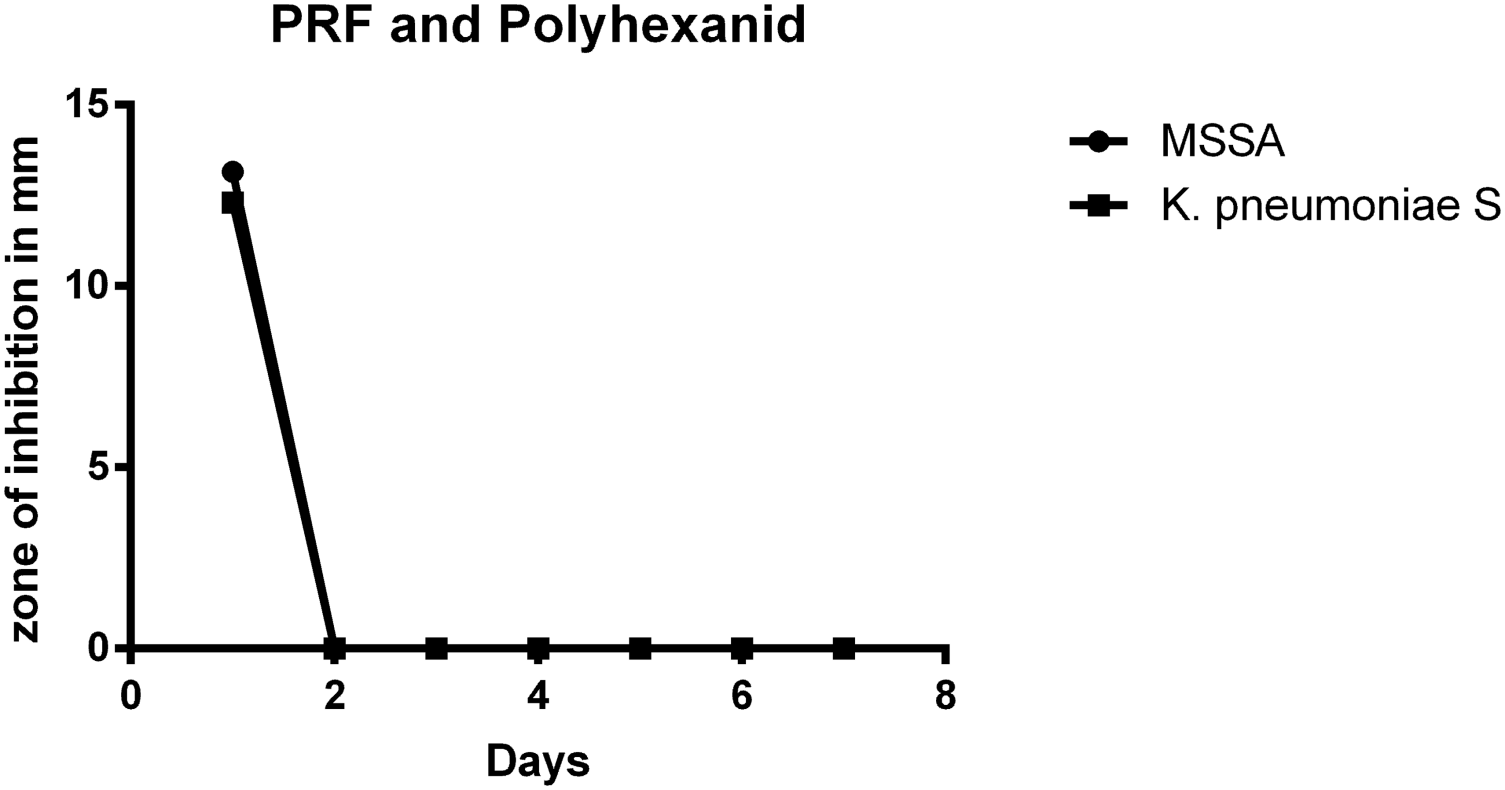
Mean zones of inhibition for MSSA and *K*. *pneumoniae* with PRF and polyhexanide. Bacterial eradication occurred within the first 24 hours only.

All bacterial strains incubated with polyhexanide showed a zone of inhibition after the first 24 hours only. Mean inhibition zone sizes were 13.17 mm (σ = 0.29 mm) for MSSA and 12.3 mm (σ = 2.31 mm) for *K*. *pneumoniae* S. *P*. *aeruginosa* had to be excluded again from evaluation of polyhexanide release kinetics because of positive PRF-only controls. Polyhexanide—release was only verifiable during the first 24 hours of incubation (Fig 5).

## Discussion

In clinical routine patients with chronic leg ulcers and PRF-therapy are often wearing their wound bandages for an entire week without changing the dressing. In patients receiving PRF plus antimicrobial as a prophylactic measure before closure of a surgical wound, only a single application is performed. Therefore, release kinetics of antimicrobials out of a PRF-layer is an important research question as it may be indicative for the antimicrobial effect of this therapy. A longer therapeutic effect provides enough time for surgical wounds to stabilize and allows a lower frequency of dressing-changes in infected wounds, thus not only improving patients´ quality of life without changing the antimicrobial effect of the therapy, but also lowering healthcare costs. [11] Although polyhexanide would be the most desirable combination partner for PRF from an antimicrobial stewardship point of view, based on our results, polyhexanide is released only during the first 24 hours of incubation, and therefore can hardly be used in wounds enclosed in a wound bandage for an entire week. Low concentrations of polyhexanide may show only limited bactericidal activity. As a consequence of the 1:1 dilution of the compound when co-delivered with PRF, a better effect may be observed when using higher polyhexanide concentrations. Albeit at the moment there is no liquid medicinal product available with more than 0.4% polyhexanide content. In contrast to polyhexanide, antimicrobial activity of teicoplanin and amikacin was observed for a period of five to seven days in bactericidal concentrations, thus allowing weekly dressing changes in infected wounds. In addition to the prolonged release, amikacin is known for its postantibiotic effect (PAE), which also applies for concentrations much lower than the MIC as shown in an *in-vitro* study for multidrug-resistant *P*. *aeruginosa*. [14] Therefore, it may be assumed that in-vivo amikacin even will show a microbicidal effect for more than five days. Unfortunately, in our study this could not be assessed, because the patch with PRF and amikacin was transferred every 24 hours to another agar with freshly smeared bacteria, which had not been exposed (i.e. affected) by amikacin prior to incubation with the patch. In our opinion, these results justify dosing at an extended interval and thereby a prolonged wearing period of wound dressings. However, it must be kept in mind that the treatment regimen chosen depends on the pathogen found in the wound. Therefore, we suggest microbiological wound testing before treatment with PRF plus antibiotic is initiated. Furthermore, a wound´s protease-activity can play a crucial role in substance release kinetics. This is because proteases degrade fibrin and thereby support release of the co-delivered antimicrobials. Unfortunately, our study has to deal with the limitation of using different patches for the delivery of antibiotic controls (Assay Discs, Schleicher & Schuell^®^) and PRF (silicon gauze, Sorbion^®^). This was inevitable as the antimicrobial substances used are too inviscid to be absorbed and subsequently released by the silicon gauze. Otherwise, application of PRF onto standard control patches (Assay discs, Schleicher & Schuell^®^) equally represented a methodological problem, because of the aggregation of PRF and its adhesion to the control patch, which would not have allowed adequate release. Furthermore, the use of silicon gauze most likely resembled the clinical situation. However, it remains to be seen whether an elevated protease-activity leads to a prolonged or abbreviated substance-release due to faster PRF-degradation or if protease-activity has no influence. Therefore, the benefit of the application of protease activity tests for prediction of antibiotic release in wounds with higher protease expression needs to be evaluated. [13] Furthermore, it has to be outlined that PRF-only showed microbicidal activity against *P*. *aeruginosa*. PRF´s antimicrobial effect is already described by Bayer *et al*. who found that PRF leads to the induction of the antimicrobial peptide human beta-defensin-2 (hBD-2) in primary keratinocytes. [15] The antimicrobial effect of platelet-derivatives has previously been described by Tohidnezhad et al., who could show, that PRP had antimicrobial effects against bacteria, such as *Escherichia coli*, *Bacterium megaterium*, *Klebsiella pneumoniae*, *Enterococcus faecalis*, and *Proteus mirabilis*. [16]PRP contains the antimicrobial peptide hBD-2, which is released by platelets. Compositely, we suspect PRF to have similar antimicrobial effects on the microbes indicated above. Nevertheless, further studies measuring hBD-2 expression levels are needed to verify these findings. [17] Another study on the antimicrobial efficacy of PRF- only is currently performed. To ascertain the clinical significance of these findings, in vivo—trials comparing PRF plus antimicrobials, PRF plus antiseptics, and PRF-only with at least one comparator arm are needed. In conclusion, our results currently cannot support the use of polyhexanide as combination partner for PRF in infected wounds. Furthermore, we suggest performing a microbial wound test prior to initiation of PRF plus antibiotic therapy to select the adequate antibiotic drug. Wound bandages in wounds treated with PRF plus amikacin or PRF plus teicoplanin can be left for at least five days regarding the antimicrobial efficacy.

## Supporting information

S1 Fig Standard curves for teicoplanin in different concentrations in presence of PRF. No synergistic effect of teicoplanin and PRF could be shown.
(TIF)

S2 Fig Standard curves for amikacin in different concentrations in presence of PRF. No synergistic effect of amikacin and PRF could be shown.
(TIF)
