# Supplementary figures and images for: In-vitro release pharmacokinetics of amikacin, teicoplanin and polyhexanide in a platelet rich fibrin—layer (PRF)—a laboratory evaluation of a modern, autologous wound treatment
Source: PLoS One. 2017 Jul 7;12(7):e0181090. doi: 10.1371/journal.pone.0181090 (PMC5501641; doi:10.1371/journal.pone.0181090)

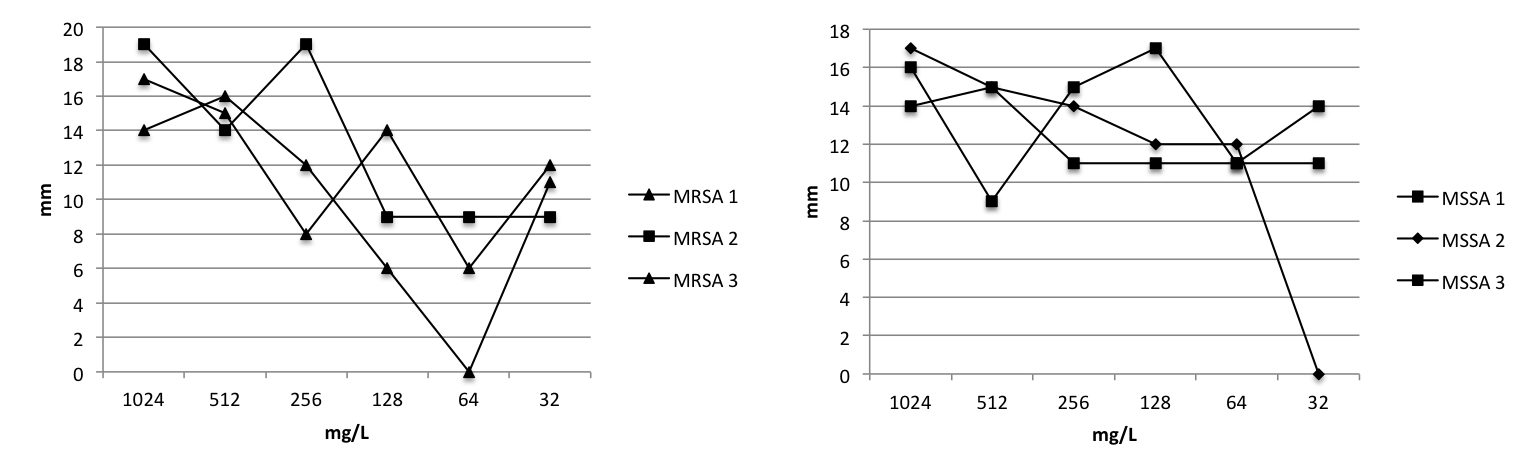

Supplement: S1 Fig — No synergistic effect of teicoplanin and PRF could be shown. (TIF) [file pone.0181090.s001.tif]

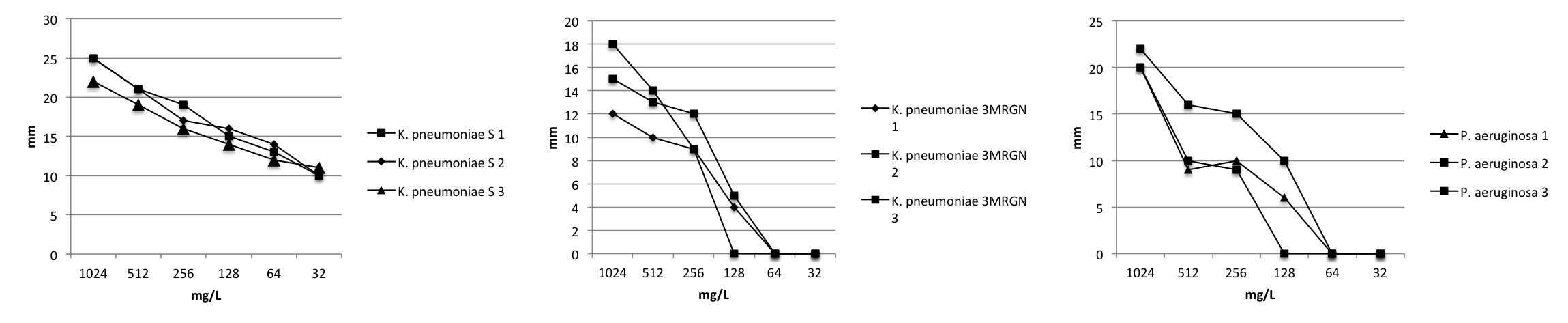

Supplement: S2 Fig — No synergistic effect of amikacin and PRF could be shown. (TIF) [file pone.0181090.s002.tif]
